# Supplementary material for: Are carbon emissions trading and green financial instruments synergistic? -Comprehensive quantitative research based on content analysis
Source: PLoS One. 2024 Mar 7;19(3):e0298601. doi: 10.1371/journal.pone.0298601 (PMC10919601; doi:10.1371/journal.pone.0298601)
Supplement: S1 Data — (ZIP) [file pone.0298601.s001.zip › Data/地级市Z_F工作报告.zip!/╔╜╢1⁄2/╝├─╧╩╨/└┤╫╘╣1⁄2╓┌║┼í╛┬φ┐╦╩2╛▌═°í┐_2013.docx]

各位代表：

　　现在，我代表市人民政府向大会报告工作，请予审议，并请各位政协委员和其他列席人员提出意见。

　　一、紧紧围绕“加快科学发展、建设美丽泉城”中心任务，凝心聚力，攻坚克难，实现本届政府工作良好开局

　　刚刚过去的一年是本届政府开局之年，是我们经受住复杂严峻经济形势考验、开启省会现代化建设新征程的关键一年。在中共济南市委坚强领导下，市政府和全市人民一起，深入学习贯彻党的十八大精神，牢牢把握主题主线和稳中求进的工作总基调，认真落实中央宏观调控政策，紧紧围绕市第十次党代会确定的“加快科学发展、建设美丽泉城”中心任务和市十五届人大一次会议提出的目标要求，解放思想，振奋精神，坚定信心，真抓实干，攻坚克难，争先进位，全市经济社会发展呈现稳中有进、和谐稳定的良好局面。

　　凝聚力量，强力推进，经济发展实现稳步回升。建立统一高效的领导推进机制，市委、市人大、市政府、市政协主要领导挂帅，共同推进发展实体经济、建设美丽泉城、优化发展环境、创新社会管理和招商引资、重点项目规划建设各项工作，制定了《全市重点工作推进实施方案》，先后召开了“加快科学发展、建设美丽泉城”推进大会和重点项目建设现场观摩会，强化调度、督查、考核和社会监督，引领形成了县（市）区竞相发展、部门创先争优的良好局面。经过全市上下坚持不懈的努力，一季度、上半年、前三季度，全市生产总值增速分别为7.5%、8.1%、8.7%，固定资产投资增速分别为11.3%、16.1%、19.4%，规模以上工业增加值增速分别为3.96%、6.11%、8.07%，地方公共财政收入增速分别为12.5%、16%、13.5%，四季度各项主要经济指标继续向好，均比前三季度有新的提高，与全省经济发展水平基本保持了同步，经济运行呈现出“低位开局、逐季向好”的态势。

　　狠抓项目、园区和招商，实体经济和开放型经济水平不断提高。全力突破薄弱环节，把项目建设作为发展实体经济、优化调整经济结构的重要支撑，实施“项目建设三年行动计划”，推进福瑞达低聚糖等296个全市重点项目建设。注重优化投资结构，引导产业结构、产品结构、技术结构、所有制结构调整，预计全年完成工业投资700亿元，同比增长21%，其中技改投入占72%；高新技术产业产值占规模以上工业总产值比重达39.55%，比年初提高1个百分点。落实鼓励和引导民间投资的实施意见，民间投资占全部投资比重达到60%左右。把园区作为发展实体经济的重要载体，加快高新区等10个省级以上开发区和20个重点服务业集聚区建设，促进产业和项目聚集。突出抓好五大区域性服务业中心发展，预计服务业增加值增长9.5%左右，金融业增加值、信息服务业收入、旅游业总收入、社会物流总额和展会交易额分别增长20%、25%、20%、12%和14%。预计年末本外币贷款比年初增加600亿元，信息服务业收入突破千亿元，社会物流总额超万亿元。出台财政、金融支持实体经济发展的意见，创新中小企业扶持机制，济南中小企业公共服务中心成为国家级服务平台，建立市级以上小企业辅导基地11家，多措并举新增“三上”企业1000家。把创新驱动作为发展实体经济的重要动力，加强科技人才引进、培育、管理，加大知识产权保护力度，新认定国家级企业技术中心3家，总数达到18家，培育省级创新型（试点）企业20家，国家超算济南中心和综合性新药研发大平台完成验收，创新型城市建设成果显著。把招商引资作为发展实体经济的重要手段，狠抓园区招商、产业招商和项目招商，精心组织参加2012香港山东周、济南（上海）投资说明会、第六届“华交会”等招商活动，引进市外资金1140亿元，增幅同比提高6个百分点。实际到账外资12.2亿美元，增长11%。对外贸易在调整中发展，高新技术产品出口增长25.7%。离岸服务外包执行额增长52.3%，居全省首位。推进明水经济开发区升级为国家级经济技术开发区，济南海关升格为总署直属海关，国家级综合保税区获批设立，为加快开放型经济发展创造了有利条件。

　　统筹城乡，分类指导，促进县区经济社会加快发展。针对区域经济发展不平衡和县域经济薄弱的突出问题，统筹城乡规划、产业布局、基础设施建设和公共服务，强化扶持政策，促进县区加快发展。继续落实举全市之力支持高新区发展和帮扶商河的各项政策，鼓励基础较好的章丘市、历城区争当跨越发展新的增长极，实施“三年突破平阴”行动计划，推动长清加快融入中心城区发展，鼓励支持其他县区抢抓机遇，发挥优势，争先进位。预计10个县（市）区和高新区，生产总值过400亿元的5个、财政收入过30亿元的3个。着力发展省会“两型”农业，重点推进农高区、现代农业示范园、都市农业园区和特色基地建设。粮食连续十年丰收，总产达到304万吨，肉蛋奶总产量连续五年突破100万吨。培育生态农产品品牌，促进农产品深加工，新认定市级农业龙头企业77家，新建畜牧业标准化生产基地30处。加强农田水利基础设施建设，新增、恢复、改善灌溉面积18.8万亩，完成6座大中型水库和126座小型病险水库除险加固以及95条小流域综合治理工程。

　　规划引领，有序推进，美丽泉城建设迈出新步伐。按照“泉城特色突出、规划布局合理、建筑和谐美观、设施配套齐全、生态环境良好、管理井然有序”的要求，全面推进城市规划建设管理工作。强化规划引领，加快编制南部绿色发展和北部跨河发展控制性规划，完成西客站核心区、滨河新区核心区城市设计，加快鹊山龙湖、济西湿地公园等项目策划。推进“一城三区”重点项目建设，老城区泉城特色标志区和商埠区改造提升步伐加快，百花洲工程初具规模，武岳庙、福慧禅林等古建筑修葺完成，启动火车站北广场规划建设，绿地普利中心、山东书城等17个综合体项目进展顺利。东部新区奥体金融中心、汉峪金融商务中心、唐冶、雪山片区建设加快。西部新区省会文化艺术中心“一院三馆”主体封顶，场站一体化工程投入使用。滨河新区小清河整治收尾工程基本完成，徐李片区和泺口涝洼地改造、非物质文化遗产博览园等项目全面展开。北部跨河发展和南部山区河道整治等重点项目有序推进。完成济齐路等42条道路建设整治，二环西路建设进展顺利。“四供两排”设施建设继续加强，新建续建35千伏及以上输变电工程43项，完成17个供水低压片区改造，新增集中供热面积700万平方米，开工建设第三生活垃圾处理厂和餐厨废弃物处理工程。开展迎“十艺节”城市环境综合整治行动，市容面貌有了较大改观。启动治理交通拥堵三年计划，城区交通综合整治初见成效。治理破损山体13座，完成造林21万亩，新建绿地4950亩、绿色通道195公里，城市绿化覆盖率、森林覆盖率分别提高0.8个和1.2个百分点。节水保泉和河道整治成效明显，群泉连续9年喷涌，大明湖及护城河水质保护工程顺利实施，省控重点河流出境断面水质基本达标，荣膺水生态文明市全国唯一试点城市。城乡环卫一体化覆盖率达到90%，提高了30个百分点，市区环境空气质量同比改善率达到2.3%。

　　强化责任，稳步提升，民生保障和社会事业实现新发展。提升公共服务，将财政支出的57.6%用于民生和社会事业重点领域，同比提高2.2个百分点。2012年为民办10件实事全面完成（附件1）。出台鼓励创业带动就业政策，解决了大学毕业生等重点群体就业2.8万人，新增城镇就业22.3万人，农村劳动力转移就业7.4万人。稳步提升社会保障水平，实现城乡居民养老保险全覆盖，农村低保标准和五保户集中供养标准分别由年均1800元、3600元提高到2300元、4200元。连续8年上调企业退休人员养老待遇，2012年人均月增269元。推行阳光民生救助体系，弱势群体社会保障救助范围不断扩大。实行全民医保，全市新农合补偿上限平均提高到14.5万元，基层公办医疗卫生机构实现了基本药物制度全覆盖。全市增加教育经费9亿元，大力推进基本公共教育服务均等化，实现公办特教全免费。投资10亿元实施普通中小学标准化建设和校舍安全工程，新建、改扩建100所幼儿园。通过校际联盟、名师交换和小区配建，教育资源布局进一步优化。提前超额完成保障性住房建设任务，开工建设公租房15524套、经济适用房3416套，竣工各类保障性安居工程7519套，工程质量在住建部执法检查中位列全国第一。扎实推进“文化惠民十大行动”，新建改建博物馆20个、基层公共文化设施415个，837个公共文化设施向市民免费开放。“十艺节”筹备工作进展顺利，儿童剧《宝贝儿》被文化部列为全国优秀保留剧目。我市运动健儿获得伦敦奥运会2金1银、残奥会1金的优异成绩。

　　提速增效，优化环境，社会管理创新取得新进展。出台《济南市人民政府关于进一步加强自身建设的意见》，重新修订《政府工作规则》。市级行政许可事项由216项精简至173项，开通重大项目行政审批绿色通道，推进行政执法电子监察系统建设和行政复议委员会试点，开展“治庸治懒治散”行动、“三服务”和机关作风建设年活动，办事效率进一步提高。自觉接受人大依法监督和政协民主监督，认真办理“南部生态经济区保护与发展”和“推进北跨战略”议案，启动规划和项目建设。办结人大代表建议402件、政协委员提案625件。健全反腐倡廉和科技防腐机制，构筑惩治和预防腐败体系。社会管理不断创新，12345市民服务热线和“阳光大姐”家政服务分别被国家标准委员会认定为全国服务标准化示范单位和标准化企业。深入推进平安济南建设，信访工作体制不断完善，社会治安保持稳定，安全生产基层基础建设和食品药品监管进一步加强。创建文明城市活动取得新进展，我市在全国城市文明程度指数测评中位列省会和副省级提名资格城市第一名。我市还荣获全国双拥模范城“七连冠”，被中国城市竞争力研究会评为中国十佳宜商城市第三名。外事、侨务、对台、口岸、民族宗教、社会科学、档案史志、广播电视、妇女儿童、慈善、红十字、老龄、残疾人、气象、人防、防震减灾等工作不断加强。

　　经过全市上下艰苦奋斗、顽强拼搏，省会现代化建设取得了新的成就：预计2012年，全市生产总值增长9.5%左右；规模以上工业增加值1357亿元，增长10%；社会消费品零售总额2320亿元，增长15%；固定资产投资2100亿元，增长20%；地方公共财政收入380.8亿元，增长17%；三次产业比例调整为5.2∶41∶53.8，城镇化率达到66%。全面完成节能减排各项约束性指标。城市居民人均可支配收入和农民人均纯收入分别达到32560元和11760元，增长12.7%和13%，均高于生产总值增长幅度。人口自然增长率为4.04‰。居民消费价格涨幅为2.4%，人民生活水平进一步提高。

　　成绩来之不易，这是中央和省委、省政府科学决策、大力支持的结果，是市委正确领导、全力推进的结果，是全市广大干部群众和社会各界团结一致、共同努力的结果。在此，我代表市人民政府，向全市各族人民，各级人大代表、政协委员、各民主党派、工商联、无党派人士、各人民团体和各界人士，向驻济人民解放军、武警官兵和中央、省驻济单位，向港澳台同胞、海外侨胞和国际友人，表示崇高的敬意和诚挚的感谢！

　　此时此刻，我们回顾过去的一年，更加深刻体会到：深入贯彻落实科学发展观是推进省会现代化建设的根本保障，必须把落实中央和省委、省政府的决策部署与济南实际紧密结合起来，解放思想，锐意进取，才能不断开创改革发展新局面；发展始终是第一要务，是解决当前面临一切问题的基础，必须坚持发展不动摇，抢抓机遇，攻坚克难，才能不断提高省会综合实力和核心竞争力；改革创新是发展的强大动力，必须善于用创新的思维、市场的办法来推进市场经济的发展，用社会的力量来解决社会面临的难题，才能更好地推动省会科学发展、和谐发展；提高质量效益是保证经济健康发展的重要支撑，必须努力转方式调结构，扩大增量消费和优化投资，对外招商引资和对内改造提升并举，才能实现稳中有进；求真务实是实现发展目标的根本途径，必须转变作风，提高效能，真抓实干，争先进位，才能确保各项工作落实到位；省会优势是我市加快发展的最大资源，必须调动方方面面的积极性，凝聚更多力量共同奋斗，才能形成推进省会建设发展的强大合力。我们对肩负的责任和压力更加清醒，对实现“加快科学发展、建设美丽泉城”的目标任务充满信心。

　　一年来的成绩令人鼓舞，但面临的困难和问题仍然不少，发展中不平衡、不协调、不可持续问题依然突出。受宏观经济形势的影响，一些骨干企业生产经营比较困难，致使生产总值、规模以上工业增加值和进出口增长趋缓；传统产业比重过大，产业结构调整任务艰巨；实体经济支撑、增量消费拉动、城乡统筹发展的能力相对较弱；就业和社会保障压力增大，部分群众生活还比较困难，一些涉及群众利益的突出问题有待进一步解决；政府职能转变还不到位，工作作风和办事效率仍需转变提高，反腐倡廉工作还要进一步加强。对于这些困难和问题，我们将在今后工作中努力解决。

　　二、牢牢把握“两个全面”的奋斗目标和“五位一体”总体布局，谋划好各项工作，促进经济社会持续健康发展

　　各位代表，党的十八大是在我国进入全面建成小康社会决定性阶段召开的一次具有里程碑意义的大会，开启了中国特色社会主义伟大事业新的征程，明确提出到2020年“全面建成小康社会和全面深化改革开放”的奋斗目标，规划了中国特色社会主义事业“五位一体”总体布局，为推进我市现代化建设新征程、率先在全省建成更高水平小康社会指明了方向。

　　2013年是全面贯彻落实党的十八大精神的开局之年，是实施“十二五”规划承上启下的关键一年，是为全面建成小康社会奠定坚实基础的重要一年。当前，经济形势依然复杂严峻，国际金融危机仍未走出困境，世界经济复苏步伐缓慢。国内经济企稳回升的基础尚不牢固，经济下行压力加大，但保持稳中有进的态势仍然具有许多积极因素和有利条件。党的十八大指出，我国总体上仍处于大有作为的重要战略机遇期，国家将继续实施积极的财政政策和稳健的货币政策，以提高质量和效益为中心，扩大内需，创新驱动，加大转方式调结构力度，稳中有进的发展环境将持续向好。从省、市看，重点区域带动战略逐步实施，实体经济推动作用日益增强，消费保持平稳增长态势，投资和出口在调整中仍有很强的拉动作用，特别是我市推出了一批深化重点领域改革和扩大开放的新举措，为“加快科学发展、建设美丽泉城”增添了新的动力和活力。

　　2013年全市经济社会发展的总体要求是：深入学习贯彻党的十八大精神，坚持以邓小平理论、“三个代表”重要思想、科学发展观为指导，牢牢把握“两个全面”的奋斗目标和“五位一体”总体布局，紧紧围绕主题主线和“加快科学发展、建设美丽泉城”中心任务，以提高经济增长的质量和效益为中心，以深化改革扩大开放为动力，着力转变经济发展方式，调整优化经济结构，强化创新驱动，推进新型工业化、信息化、城镇化、农业现代化同步发展，增强经济综合实力和文化软实力，提高生态文明建设水平，切实保障和改善民生，促进经济持续健康发展和社会和谐稳定进步。

　　综合考虑国内外经济形势和济南发展实际，2013年经济社会发展的主要预期目标是：生产总值增长9.5%以上，地方公共财政收入增长13%，固定资产投资增长18%，社会消费品零售总额增长15%。服务业增加值增长10%以上，规模以上工业增加值增长10%以上，高新技术产业产值比重提高1个百分点。外贸出口增长8%，实际到账外资增长8%。城市居民人均可支配收入、农民人均纯收入分别增长11%和12%，居民消费价格总水平涨幅控制在3.5%左右，城镇登记失业率控制在4%以内，人口自然增长率控制在5.5‰以内，完成省政府下达的年度节能减排目标任务。重点抓好以下工作：

　　（一）努力推动结构调整和实体经济发展。牢牢把握扩大内需这一战略基点，贯彻落实国家宏观调控政策，以提高质量效益和增加就业为重点，以存量强根基，以增量促调整，加快结构调整和发展方式转变，把推动发展的着力点转到培育形成新活力、新动力、新体系、新优势上来，打造省会经济新的增长点。

　　推动经济持续健康发展。切实增强消费对经济增长的基础作用，积极扩大消费需求，促进城乡居民收入增长，提高居民消费能力和信心。扩大文化娱乐、旅游休闲、家政服务、社会养老、医疗保健、体育健身等消费增量。加大设施投入，扎实推进重点文化旅游项目建设，深化“万村千乡市场工程”和“农超对接”工程，抓好肉菜追溯、家政服务和养老服务等体系建设。充分发挥投资对经济增长的关键作用，保持投资稳定增长，不断优化投资结构，促进招商引资向招商选资转变，争取现代服务业和战略性新兴产业投资比重达到80%左右，民间投资比重稳步提高。注重提高经济增长的质量和效益，既要看当前，更要看长远，上项目要上有质量、有效益、能够增加就业的项目，绝不能上落后产能和产能过剩项目。按照“四抓”的要求搞好重大项目评估和飞地项目认定等工作，建立完善重点项目年度用地指标竞争分配机制，全面落实“项目建设三年行动计划”，着力推进213个重点项目建设。抓好财税工作，加大财源建设力度，深化财税体制改革，调整完善财政管理机制。保持物价总水平基本稳定。组织好第三次全国经济普查工作。

　　做大做强工业经济。进一步优化“一高三区”工业布局，以园区、项目、产业为重点，推进信息化与工业化深度融合。加快省级以上开发区转型发展，按照每个开发区规划发展2-3个主导产业的要求，编制完善全市开发区空间和产业规划，促进各类生产要素集聚重组。优先保障开发区重点项目建设用地，吸引优势产业和项目向园区集中，力争用3年时间实现省级以上开发区经济总量翻一番，主要指标增幅实现“三高一进位”。健全项目协调推进机制，着力破解资金、土地、征地拆迁等难题，确保民基电力、阿胶产业园、爱之味食品等75个投资244亿元的重点项目按期建成。深入推进“双轮驱动”战略，加快发展天岳晶体材料、华云豪克能机床等一批成长型企业，战略性新兴产业销售收入增长25%以上。实施中小企业成长计划，推动“五个一批”梯度培育工程，鼓励支持中小微企业向“专精特新”发展。加快传统产业改造升级，争创传统产业竞争新优势。加强“智慧泉城”和“两化”融合试点城市建设，提升城市综合信息服务能力，打造更高水平中国软件名城。加大齐鲁软件园、济南“创新谷”、历下高端信息服务业基地建设，形成“一园一谷一基地”的高端产业布局。实施创新驱动发展战略，推进国家创新型试点城市和国家综合性高技术产业基地建设，力争新认定市级以上企业技术中心20家，抓好国家重大新药创制平台、量子技术研究院等重大源头创新平台建设，加快云计算、高效能服务器、生物化工等关键技术研发和产业化，加大知识产权保护利用，推动创新型经济快速成长。提升“5150引才倍增计划”，贯彻落实“科技11条”政策，鼓励各类人才创业和科技成果转化。尊重企业主体地位，注重发挥企业家才能，促进产品创新、品牌创新、产业组织创新和商业模式创新。

　　推进服务业跨越发展。深化国家服务业综合改革试点，推动服务业扩大规模、优化结构、提升层次、增加效益。加快区域性金融中心建设，做好济南金融商务中心区综合改革试点工作，建立山东省金融资产交易中心、企业上市路演中心、金融数据中心，促进安顾寿险、鲁银投资等项目的落地，积极发展小额贷款公司、担保公司、村镇银行等金融组织。支持高新区进入全国代办试点范围，争取2-3家企业上市融资。做好金融监管工作，防控金融风险。推动旅游业加快发展，完善国际旅游名城创建规划，培育大型旅游企业集团和一批著名旅游景区，加快南部山区旅游集散中心、平阴龙冈梦幻谷、章丘砂之船艺术商业广场等重点项目建设，做好“天下第一泉”5A级景区创建和“泉水节”筹办工作，推进文化旅游产品开发，提高旅游服务水平。大力发展现代物流业，加强物流园区规划建设，推进翰迪物流园二期、重汽配件物流中心、豪诺医药物流等重点项目建设，推广电子商务等现代流通方式，加快构建现代流通体系。支持会展业更好发展，建设西城会展中心，申办一批效益好、档次高的国字号展会。坚决贯彻落实房地产市场调控政策，优化供应结构，满足居民合理住房需求，引导房地产业健康稳定发展。以国家住宅产业化综合试点城市为契机，加快新兴住宅工业化发展。提升批零贸易、住宿餐饮、交通运输、居民服务等产业，培育工业设计、科技咨询、节能环保等新型业态，规划建设总部基地，壮大以总部经济、楼宇经济为主的城区服务经济规模。各县（市）区都要筹建培育1-3处高水平特色商业街区和在全省有影响、辐射周边的区域专业市场，打造平台，促进民营经济发展壮大。

　　提升农业现代化水平。巩固农业经济基础地位，推进农业经营机制创新，构建省会现代农业发展新框架，确保农业增产、农民增收。实施30万吨粮食增产规划，力争连续丰产丰收，产量稳定在300万吨左右，确保粮食安全。加快推进蔬菜、畜牧、林果、种苗花卉产业振兴规划，建设50万亩城市保供菜田。发展多种形式的适度规模经营，通过抓龙头、拓市场、建基地、联农户，实现“四位一体”同步推进，新认定市级农业龙头企业50家、合作社示范社100家、畜禽标准化生产示范基地20家。加强科技兴农，搞好技术推广和品牌创建，推进市农高区、6个县级农业科技示范园、200个都市农业园区、50个特色品牌基地和20个现代农业示范乡镇建设。加快4个小型农田水利重点县、2个高效节水灌溉试点县建设，建成30万亩高标准农田，解决20万农村居民饮水安全问题。落实强农富农惠农政策，积极发展农村二三产业，深化农村集体资产改革，加快农村现代经营服务体系建设，增加农民非农收入和财产性收入。深入推进农村扶贫开发，实现6万贫困人口人均增收1100元的目标。

　　（二）促进城乡融合发展，积极推进城镇化进程。全面提升城镇规划建设管理水平，加大城乡统筹力度，加快县区经济发展，让城镇更好地承载发展、造福全市人民。

　　深入推进美丽泉城建设。提升城市功能形象，拓展优化中心城区空间布局。老城区重点抓好商埠区、百花洲片区、解放阁世茂广场等项目建设，着力打造服务经济发达、历史文化彰显的现代化中心城区。东部新区推动奥体文博、唐冶、雪山等重点片区开发建设。西部新区加快恒大国际金融中心等项目规划建设，确保省会文化艺术中心“一院三馆”、站前综合体、公交枢纽、长运枢纽和东广场按期投入使用。滨河新区重点搞好核心区和吴家堡片区规划建设，全面启动北湖片区和通航河道工程。在“一城三区”开发建设中，注重古城区保护性提升，积极破解难题，加快棚户区、城中村和危旧楼群改造。继续搞好重大基础设施建设，启动轨道交通建设，开工二环西路高架南延、顺河高架路南延与二环南路高架工程，加快山大路、纬十二路等一批路桥工程和济南火车站北广场建设。继续推进城市交通综合整治三年行动计划，加快建设“公交都市”。搞好天然气利用规划，推进供水、供热、供电、生活废弃物处理设施和第三垃圾处理厂建设，提高市政公用设施服务覆盖率。加强城市环境综合整治，抓好市容道路、城市出入口、城乡结合部、铁路沿线等重点部位环境整治，集中解决占道经营、违法违章建设等城管难点，为市民营造更加整洁靓丽的城市环境。

　　提升县区经济社会发展水平。鼓励强市强区加压奋进，支持欠发达县区努力赶超。历下、市中、槐荫、天桥等中心城区，要加快打造金融、商贸、信息服务等现代服务业集聚发展的新高地。历城、章丘要重点抓好先进装备制造业、临港工业和现代物流业发展。长清、平阴、济阳、商河等县区，要用好扶持政策，重点抓好济北开发区、“创新谷”、平阴百亿产业集群、商河温泉国际等园区和产业发展项目建设。强化产业支撑、项目带动，加快推进南部绿色发展和北部跨河发展，培育县域经济发展新的增长点。

　　积极稳妥推进城镇化。以“提质加速、城乡一体”为目标，规划引领，统筹推进，着力提高城镇化质量和水平，力争城镇化率提高1个百分点以上。科学规划城镇规模布局，提升城镇综合承载能力，推进省级、市级示范镇建设，启动3万户50个城中村改造项目，规范有序建设新型农村社区。深化户籍、土地管理制度改革，研究制定有关社会保障政策，有序推进农业转移人口市民化。

加快建设省会城市群经济圈。按照省委、省政府关于省会城市群经济圈发展规划，落实好强化省会城市核心地位、建立区域一体化互动协调机制、加大资源要素整合力度的部署要求，与周边城市优势互补、携手发展。

　　（三）以迎办“十艺节”为契机，促进文化大发展大繁荣。发挥我市文化资源富集的优势，推进社会主义文化建设。

　　全面做好“十艺节”筹办工作。精心承办“文华奖”、“群星奖”等各类艺术赛事，提升重点参赛剧目水平，打造一批在全国有影响的艺术作品。搞好社会动员，营造良好氛围，做好接待服务、综合保障、治安保卫等各项工作。

　　繁荣发展文化事业。重点推进文化惠民工程，开展市第三届民俗文化艺术节、第四届齐鲁民间艺术表演、“书韵泉城”全民阅读节、“泉城大舞台”等活动。加强文化对外交流合作，鼓励社会力量积极参与公共文化项目建设，编制实施《省会百座博物馆建设规划》，保护传承文化遗产，重点抓好大遗址保护、齐长城（济南段）文化景观带和重点文物保护三大工程。

　　提升文化产业竞争力。促进文化与科技、金融、教育、旅游融合发展，鼓励发展文化创意、数字出版、动漫游戏等新兴产业，推动媒体业、印刷业等传统产业转型升级。高水平规划建设非物质文化遗产博览园、老商埠文化休闲商务区，提升英雄山文化市场效益和水平。实施规模以上文化企业增量工程，支持41个过亿元骨干企业做大做强。挖掘打造龙山文化、泉水文化、名士文化、商埠文化、曲山艺海等特色文化品牌，探索“十艺节”文化载体市场化、商业化运作模式。

　　积极创建全国文明城市。加强领导，精心组织，深入动员，突出实效。加强社会主义核心价值体系建设，开展济南解放65周年纪念活动，激励广大市民爱家乡、作奉献，弘扬优秀传统文化和“诚信、创新、和谐”济南城市精神，提升城市文明程度和市民文明素质。深化群众性精神文明创建活动，鼓励支持“泉城义工”志愿服务行动，广泛开展科普活动。加强文化市场综合执法和网络管理，营造健康有序的社会文化环境。

　　（四）改善民生和创新管理，全面加强社会建设。积极回应人民群众对美好生活的新期待，以保障和改善民生为重点，加强和创新社会管理，推进社会建设取得新进展。完成今年确定的17件民生实事（附件2）。

　　促进就业稳定增长。就业是民生之本，创业是就业之源。实施就业优先战略和更加积极的就业政策，促进创业带动就业，做好以高校毕业生为重点的青年就业和农村转移劳动力、城镇困难人员、退役军人、被征地人员就业工作。全年新增城镇就业10万人，农村劳动力转移就业5.2万人。

　　统筹推进城乡社会保障体系建设。进一步扩大社会保险覆盖面，落实职工养老保险、城乡居民养老保险衔接转换政策，加快阳光民生救助体系建设，完善困难群体物价上涨补贴联动机制，逐步提高城乡低保、农村五保、城镇“三无”人员保障和孤儿养育标准，健全社会养老服务体系，大力发展养老服务业，完成市养老服务中心建设一期工程。

　　努力办好人民满意的教育。实施县域义务教育均衡发展规划，适度扩大外来务工人员子女学校数量，实施骨干教师跨校交换制度，促进优质教育资源向农村、城乡结合部、新建小区扩展。完成学前教育三年行动计划和校舍安全工程五年规划任务，公办幼儿园和在园幼儿比例均达到65%。加快推进普通教育高中与职业学校资源共享、融合发展。

　　提升医疗为民服务水平。健全卫生保健服务体系，实行城镇居民医疗保险门诊统筹，新农合人均筹资标准提高到350元以上。建立重特大疾病保障和救助机制，完善基本药物制度体系，继续推进县级公立医院综合改革试点工作，鼓励社会办医，启动基层中医药服务能力提升工程，加快市中心医院综合楼等重点项目建设。

　　加大保障性安居工程建设力度。重视解决人才公租房和中等偏下收入住房困难家庭及新就业人员、外来务工人员的基本居住问题。建设保障性安居工程1.5万套，提供分配保障性住房不少于6000套（间），完成农村危旧房改造2500户。

　　加强和创新社会管理。加强基层社会管理和服务体系建设，增强城乡社区服务功能。引导社会组织健康有序发展，充分发挥群众参与社会管理的基础作用。深入推进平安济南、法治济南建设，全面实施“六五”普法规划和“五五”依法治市纲要，依法防范和严厉打击各类违法犯罪活动，健全信访工作责任制，维护社会和谐稳定。高度重视安全生产，强化道路交通、烟花爆竹、消防、校园安全监管，防止重特大事故发生。加强市场监管，规范市场秩序，完善食品药品安全监管机制。加强地震、气象等防灾减灾体系建设，确保城市安全运行。继续做好人口计生工作，提高出生人口素质。大力发展老龄服务和慈善事业，发挥好红十字会作用，健全残疾人社会保障和服务体系，加强民族宗教、档案史志工作。大力推动全民健身运动，提高竞技体育水平。推进国防动员、后备力量和人民防空建设，做好双拥优抚安置工作。

　　（五）努力创建生态文明城市，构建省会永续发展新格局。把生态文明建设放在更加突出的地位，统筹抓好生态城市、水生态文明市、生态园林城市、森林城市、环保模范城“五城联创”。

　　搞好水生态文明创建。编制完善水生态文明市创建规划和实施方案，推进三大重点片区生态水系工程，实施多水源连通、东部工业区地下水置换、玉符河综合整治等项目。加大措施保证泉水持续喷涌。完成城区48条河道截污整治，实现城区河道污水不直排。

　　提高城乡绿化水平。统筹国家生态园林城市和森林城市创建工作，实施规划扩绿、工程造绿、见缝插绿、社会增绿、均衡布绿、标准建绿，突出城区出入口、城乡结合部、国道、省道、高速公路、河道水系及可视荒山绿化美化，建设提升绿地150万平方米。新增造林面积20万亩，森林覆盖率提高1个百分点以上。

　　抓好大气污染防治。制定黄标车限行管理措施，防治扬尘污染，加大PM2.5整治力度。深入开展创建生态城市和环保模范城市活动，抓好章丘、商河、平阴、济阳创建国家和省级生态县（市）工作。

　　推进节能减排工作。实施重点领域节能攻坚工程，加快既有建筑节能改造，确保100户重点用能企业累计完成节能量105万吨标准煤。大力发展循环经济，培育节能低碳产业和新能源、可再生能源产业，加快推进大唐风电平阴二期和长清一期投产并网，支持力诺等太阳能光伏发电项目发展。严格落实污染物排放总量前置审核制度，抓好重点行业、企业污染物减排工作。

　　三、深化改革扩大开放，加强政府能力和作风建设，全面增强经济社会发展的动力和活力

　　深入贯彻落实党的十八大精神，解放思想，改革开放，全面提升政府行政效能，为经济社会发展提供体制机制保障。

　　（一）进一步解放思想提升能力。牢固树立科学发展理念，勇于冲破陈旧思想观念的束缚，不断提升境界，提高标准，强化率先发展、争先进位意识，不畏难、不怕苦、不躲避，敢于负责，勇于担当，真正把心思和精力集中到干事创业上，把满腔热情倾注到为民造福上，积极进取，奋发有为。正确认识工作中存在的困难和问题，努力从境界标准上找差距，从能力水平上找不足。积极推进学习型、创新型、服务型政府建设，把学习成果转化为谋划发展的思路、攻坚克难的措施和解决问题的本领，不断增强推动科学发展的能力。

　　（二）扎实推进各项改革。落实重点领域改革方案。市委、市政府已经出台了《关于深化全市重点领域改革和扩大开放的意见》及十项实施方案，重点推进行政审批改革、科技创新、文化改革、金融业发展、招商引资体制机制创新、总部经济发展、城镇化、民营经济发展、省级以上开发区转型升级跨越发展和提高开放型经济水平。这是我市贯彻落实十八大精神的重要举措，也是促进经济持续健康发展的制度保障。要妥善处理整体设计与分步实施、全面推进与把握重点、先行先试与循序渐进的关系，实现改革力度、推进速度与实际效果的有机统一。改革实施主体要主动做好对接和承接工作，确保改革开放的政策措施落到实处。通过深化重点领域改革，处理好政府和市场的关系，建立起精简高效的行政体制、科学有效的管理体制、规范有序的市场机制、公平公正的政策体系。继续深化国有企业改革，进一步增强国有经济活力和影响力。深化投融资体制改革，增强市属四大投资集团融资、运营和可持续发展能力。推动资源性产品价格改革，形成公共资源出让收益合理共享机制。探索市政服务多元化改革，更好地满足人民群众对供水、供热、供气的需求。深化行政管理体制改革，分类推进事业单位改革。

　　（三）全面扩大对外开放。坚定不移地实施全方位开放战略，进一步更新理念，增强意识，营造氛围，夯实基础，推动我市对外开放朝着优化结构、拓展领域、提高效益方向转变。积极发展对外贸易，抓好机电产品出口基地、科技出口创新基地、省级外贸转型升级示范基地和出口农产品质量安全示范区建设，鼓励企业充分利用海关特殊监管区发展出口贸易，提高机电产品、高新技术产品和高附加值农产品出口比重。搞好综合保税区规划、招商和设施建设，确保按期封关运行。加快打造中国服务外包名城，力争服务外包离岸执行额突破8亿美元。提高利用外资质量，开展“外商投资环境提升年”活动，积极推进外资并购、企业境外上市和私募股权基金等利用外资方式，抓好与新加坡合作的新济科技服务园、与欧洲易赛合作的中欧可持续发展产业园等园区建设。加强对外合作交流，充分发挥外事、侨务、对台、贸促等部门的作用，加大对港澳台地区、重点国家和世界500强企业招商引资力度，鼓励国外跨国公司在我市设立分支机构。加快我市企业“走出去”步伐，参与境外资源开发和境外工程承包，带动成套设备及零部件出口，培育一批有竞争力的跨国公司。提高口岸开放水平，促进开放型经济更好发展。

　　（四）改进政府工作作风。坚决执行中央和省、市改进工作作风、密切联系群众的规定要求，以群众为根本，以实践为标准，加强作风建设，提高服务效能。坚持科学民主决策，凡属重大事项和重要民生问题，都要深入调查研究，广泛征求意见。完善重点工作推进体制机制，加强协调，高效运转。坚持求真务实，力戒形式主义，一切从实际出发，深入基层，深入群众，多做打基础、利长远的事，针对薄弱环节，实施重点突破，解难题、办实事、求实效。密切联系群众，加快市政务服务中心建设，办好12345市民服务热线，更好地问政于民、问需于民，服务群众、方便群众。倡导艰苦奋斗、勤俭办事，力戒铺张浪费，精减会议和文件，进一步压减“三公”支出。坚持依法行政，建设法治政府，加大信息公开力度，自觉接受人大法律监督、政协民主监督和社会舆论监督，充分听取各民主党派、工商联、无党派人士和各人民团体的意见，加强对行政权力的监督制约。加快推进惩治和预防腐败体系建设，深入开展以为民务实清廉为主要内容的党的群众路线教育实践活动，强化效能监察和审计监督，加大行政问责力度，严肃查处各类违法违纪案件，切实做到干部清正、政府清廉。

　　各位代表！新的征程任重道远，新的目标催人奋进。让我们紧密团结在以习近平同志为总书记的党中央周围，高举中国特色社会主义伟大旗帜，全面贯彻落实党的十八大精神，在中共济南市委的坚强领导下，坚定信心，凝聚力量，奋力拼搏，攻坚克难，为率先建成更高水平小康社会、创造省会更加幸福美好的明天作出新的更大贡献！
